# Fine Mapping and Transcriptome Analysis of Virescent Leaf Gene *v-2* in Cucumber (*Cucumis sativus* L.)

**DOI:** 10.3389/fpls.2020.570817

**Published:** 2020-09-25

**Authors:** Kaijing Zhang, Ying Li, Wenwei Zhu, Yifan Wei, Martin Kagiki Njogu, Qunfeng Lou, Ji Li, Jinfeng Chen

**Affiliations:** ^1^National Key Laboratory of Crop Genetics and Germplasm Enhancement, College of Horticulture, Nanjing Agricultural University, Nanjing, China; ^2^College of Agriculture, Anhui Science and Technology University, Fengyang, China; ^3^Nanjing Vegetable Science Research Institute, Nanjing, China

**Keywords:** *Cucumis sativus* L., leaf color mutant, virescent leaf, auxin signaling transduction, auxin F-box protein

## Abstract

Leaf color mutants are the ideal materials to explore the pathways of chlorophyll metabolism, chloroplast development and photosynthesis system. In this study, a new virescent leaf mutant 104Y was identified by spontaneous mutation, whose cotyledon and upper five true leaves were yellow color. The yellow true leaves gradually turned green from top to bottom with increased chlorophyll contents. Genetic analysis indicated that the virescent leaf was controlled by one single recessive gene *v-2*, which was accurately mapped into 36.0–39.7 Mb interval on chromosome 3 by using BSA-seq and linkage analysis. Fine mapping analysis further narrowed *v-2* into 73-kb genomic region including eight genes with BC_1_ and F_2_ populations. Through BSA-seq and cDNA sequencing analysis, only one nonsynonymous mutation existed in the *Csa3G890020* gene encoding auxin F-box protein was identified, which was predicted as the candidate gene controlling virescent leaf. Comparative transcriptome analysis and quantitative real-time PCR analysis revealed that the expression level of *Csa3G890020* was not changed between EC1 and 104Y. However, RNA-seq analysis identified that the key genes involved in chlorophyll biosynthesis and auxin signaling transduction network were mainly down-regulated in 104Y compared with EC1, which indicated that the regulatory functions of *Csa3G890020* could be performed at post-transcriptional level rather than transcriptional level. This is the first report to map-based clone an auxin F-box protein gene related to virescent leaf in cucumber. The results will exhibit a new insight into the chlorophyll biosynthesis regulated by auxin signaling transduction network.

## Introduction

In higher plants, leaf color variation occurs commonly at different stages of growth, which is usually caused by the mutations of some critical genes related to the chloroplast development, chlorophyll biosynthesis and plant photosynthesis (Mao et al., 2019). For examples, the mutation of chloroplast ribosomal protein L21 displays an embryo-lethal phenotype in *Arabidopsis thaliana* (Yin et al., 2012). The mutations of pentatricopeptide repeat (PPR) proteins involved in chloroplast RNA editing lead to abnormal seedling color in *Arabidopsis thaliana* and rice (Zhou et al., 2009; Su et al., 2012). The *Arabidopsis VAR2* gene, encoding for FtsH2 protein, is involved in photosystem II (PSII) repair and degradation of unassembled Fe-S proteins of the cytochrome *b6f* complex, and the *var2* mutant shows green and white sectors in leaves (Chen et al., 1999; Chen et al., 2000; Takechi et al., 2000). Except for the chloroplast development, chlorophyll biosynthesis and photosynthesis pathway, many hormones are also involved in light-induced seedling greening. Liu et al. (2017) summarized a regulatory network of chlorophyll biosynthesis regulated by plant hormones including auxin, ethylene, cytokinin, gibberellin and so on. For instances, Kobayashi et al. (2017) reported that chlorophyll synthesis genes are markedly activated in detached roots *via* cytokinin but are repressed by auxin, suggesting that auxin signaling is involved in the regulation of chlorophyll biosynthesis in the root greening response. Ethylene could dramatically represses Pchlide accumulation and induces the gene expressions of both PORA and PORB in etiolated seedlings, ultimately affecting chlorophyll biosynthesis (Zhong et al., 2009; Zhong et al., 2010; Zhong et al., 2014).

A series of leaf color variations have been reported, which included albino, xanthan, light green, virescent, stripes, zebra and stay-green (Khan et al., 2005; Park et al., 2007). Because of the ease of recognition, numerous leaf color mutants have been identified and characterized in many plant species such as *Arabidopsis thaliana* (Kobayashi et al., 2008; Huang and Li, 2009), rice (Dong et al., 2013; Deng et al., 2014), wheat (Zhang et al., 2017), maize (Sawers et al., 2006), cabbage (Liu et al., 2016), pepper (Barry et al., 2008) and carrot (Nothnagel and Straka, 2003). For example, in rice, at least 208 leaf color mutants have been reported. Of those, 175 mutants have been analyzed and 154 mutant genes have been mapped on all 12 chromosomes (Deng et al., 2014). Leaf color mutations affected abnormal chloroplast development (Sun et al., 2017), abnormal chlorophyll synthesis (Mao et al., 2018), altered photosynthetic capacity (Sang et al., 2010) and delayed aging (Wang et al., 2018). These leaf color mutants are the ideal materials to investigate the molecular mechanisms of chloroplast development, chlorophyll biosynthesis and plant photosynthesis (Stern et al., 2004).

Virescent leaf is a specific and important type of leaf color mutation, which shows light yellow cotyledon or true leaf at an early stage and gradually turns green during leaf development. Most of virescent leaf mutants are thermo-sensitive or light-sensitive (Archer and Bonnett, 1987; Yoo et al., 2009). A number of virescent leaf mutants have been characterized in some species including barley (Maclachlan and Zalik, 1963), common bean (Dale and Heyes, 1970), cotton (Benedict et al., 1972), peanut (Benedict and Ketring, 1972), maize (Hopkins and Elfman, 1984; Langdale et al., 1987), *Arabidopsis thaliana* (Brusslan and Tobin, 1995; Koussevitzky et al., 2007), rice (Sugimoto et al., 2007) and so on. Many virescent leaf genes have been positional cloned in these plants. For examples, five rice virescent mutant genes have been cloned including *V1* (Kusumi et al., 2011), *V2* (Sugimoto et al., 2004; Sugimoto et al., 2007), *V3* (Yoo et al., 2009), *YSA* (Su et al., 2012) and *v14* (Zhang et al., 2014). Xing et al. (2014) identified a virescent yellow-like (*vyl*) maize mutant and validated one of the proteolytic subunits of the chloroplast Clp protease complex (*Chr.9_ClpP5* gene) as the candidate gene for *vyl*. These studies have provided ideal tools for understanding the molecular mechanisms of virescent leaf mutations and laid the foundation for applying the virescent leaf genes to plant breeding.

Cucumber, *Cucumis sativus* L. (2n = 2x =14), is a popular vegetable crop around the world. Although 17 leaf color mutants including *light green cotyledons-1* (*lg-1*), *light green cotyledons-2* (*lg-2*), *yellow cotyledons-1* (*yc-1*), *yellow cotyledons-2* (*yc-2*), *golden leaf* (*g*), *virescent* (*v*), *virescent-1* (*v-1*), *variegated virescent* (*vvi*), *yellow plant* (*yp*), *yellow-green leaf* (*yg1*), *virescent-yellow leaf* (*vyl*), *yellow young leaf-1* (*yyl-1*), *chlorophyll deficient* (*cd*), *golden cotyledon* (*gc*), *albino cotyledon* (*al*), *light sensitive* (*ls*) and *pale lethal* (*pl*) (Weng and Wehner, 2017; Song et al., 2018; Ding et al., 2019; Hu et al., 2020) have been identified in cucumber, however, limited work has been conducted on genetic mapping of these leaf color mutant genes or elucidating their regulation mechanisms. Up to date, only five leaf color mutant genes have been map-based cloned. For instances, Gao et al. (2016) reported that the chlorophyll-deficient golden leaf mutation in C528 was due to a single nucleotide substitution in *CsChlI* for magnesium chelatase I subunit. Miao et al. (2016) conducted the genetic mapping of the *v-1* locus in the virescent mutant 9110Gt, and considered that *CsaCNGCs* on chromosome 6 was the candidate gene conferring for virescent color in 9110Gt. Song et al. (2018) reported a virescent-yellow leaf (*vyl*) mutant derived from an EMS-mutagenized, and confirmed that gene *Csa4G637110* on chromosome 4 was the most likely candidate gene for *vyl*. Ding et al. (2019) reported that the formation of *ygl* mutant in cucumber was caused by the mutation of tandem *13-lipoxygenase* (*13-LOX*) genes in a cluster. Hu et al. (2020) revealed that a mutation in *CsHD* encoding a histidine and aspartic acid domain containing protein leaded to the formation of *yyl-1* mutant in cucumber.

In this study, a new cucumber virescent leaf mutant 104Y was identified by natural mutation, which exhibited stable virescent leaf phenotype, dwarf but fertile. Genetic analysis with BC_1_ and F_2_ populations derived from the cross between EC1 and 104Y indicated that the virescent leaf was controlled by one single recessive nuclear gene. According to the international rules for the naming of cucumber genes, and the *virescent* (*v*) and *virescent-1* (*v-1*) have been named (Miao et al., 2016; Weng and Wehner, 2017), thus, the virescent leaf mutant gene in this study was named as *virescent-2* (*v-2*). By combining BSA-seq (bulked segregant analysis and next generation sequencing) with linkage analysis, a candidate gene *Csa3G890020* encoding an auxin F-box protein was map-based cloned. Furthermore, comparative transcriptome analysis between the first leaves of EC1 and 104Y revealed that the chlorophyll biosynthesis could be regulated by auxin signaling transduction pathway. This study will provide new insights into understanding the virescent leaf mutation.

## Materials and Methods

### Plant Materials and Phenotypic Data Collection

The virescent mutant 104Y was self-pollinated for six generations, which exhibited the stable phenotype of virescent trait. The green cucumber inbred line EC1 was selected as maternal parent because of the significant phenotypic difference with 104Y. The F_1_, F_2_, and BC_1_ populations were subsequently generated to analyze the inheritance pattern of virescent gene (*v-2*) and map-based clone the *v-2* locus. A total of 185 F_2_-A plants were used for BSA-seq analysis and linkage analysis of *v-2* locus. 249 and 108 virescent plants from F_2_-B and BC_1_-A populations respectively, and 686 F_2_-C individuals were used for fine mapping of *v-2* locus. The phenotypic data on leaf color were collected at the seedling stage because of the easy identification of leaf color between yellow and green. The segregation ratios in the F_2_ and BC_1_ populations were analyzed with a *χ*^2^ goodness of fit test using SPSS software.

### Measurement of Pigment Contents

The contents of Chlorophyll a (Chla), Chlorophyll b (Chlb) and total Chlorophyll (Chl) were measured with the leaves of virescent mutant 104Y and green cucumber plant EC1 at five different leaf positions. The fresh leaves from the first true leaf to the fifth true leaf in 104Y and EC1 were picked and cut into pieces. For chlorophyll extraction, 0.2 g leaves was put into 50 mL tubes with 10 mL acetone-ethanol-distilled water mixture (volume ratio 4.5:4.5:1) in the dark for two days until the leaves turned white. The supernatants were collected by centrifugation and were analyzed for Chla and Chlb contents using an ultraviolet-visible spectrophotometer (UV-1100, Shanghai MAPADA Instruments Co., Ltd., China) at absorbance values of 645 and 663 nm, respectively, following the methods of Tang et al. (2004). Chla, Chlb and Chl were calculated according to the following formula:

Chla=(12.7×OD663-2.69×OD645)×V/(1000×W)

Chlb=(22.9×OD645-4.68×OD633)×V/(1000×W)

Chl=(20.21×OD645+8.02×OD633)×V/(1000×W)

V: the volume of the extraction solution, W: the weight of the sample.

### Transmission Electron Microscopy Observation

Since the color of first true leaves between 104Y and EC1 were significantly different, the first true leaves of 104Y and EC1 were used as the samples to perform the transmission electron microscopy (TEM) observation. Transverse sections of the leaf samples were fixed in 2.5% glutaraldehyde in a phosphate buffer at 4°C for 4 h. Tissues were stained with uranyl acetate, dehydrated in a gradient ethanol series and embedded in Spur’s medium prior to ultrathin sectioning. The samples were air dried, stained again, and visualized with a Hitachi S-3500N scanning electron microscope.

### BSA-Seq Analysis of *v-2* Locus

For BSA-seq analysis, virescent and green bulks were prepared from 185 F_2_-A plants based on the precise phenotypic collection. The isolated genomic DNA from 21 of each virescent and green F_2_ individuals were pooled together with an equal amount to constitute virescent bulk (A-bulk) and green bulk (B-bulk), respectively. Thus, virescent bulk, green bulk and two parental bulks (104Y and EC1) were constructed as pair-end sequencing libraries and sequenced individually on Illumina Hiseq 2500 (Illumina lnc., San Diego, CA, USA) NGS platform. To acquire the reference-based assembly, the filtered high-quality sequences (Q-score of 30 > 90%) obtained from EC1 were mapped and aligned against the reference cucumber genome sequence (ChiniseLong_V2) using BWA (0.7.12-r1039) (MEM procedure) with default parameters. Reads of virescent and green bulks were respectively aligned to EC1 assembly sequence reads to call SNPs using GATK software (McKenna et al., 2010). The called SNPs were aligned to the re-sequencing data of 104Y to confirm the genomic region associated with *v-2*.

SNP index for both bulks was calculated by comparing with the EC1 assembly following the formula suggested by Abe et al. (2012). The SNP positions with read depth < 7 in both bulks and SNP-index < 0.3 in either of the bulks were filtered out. SNP-index for each SNP position in both bulks was calculated using the following formula: SNP-index (at one position) = count of alternate base/count of reads aligned (Abe et al., 2012). Δ (SNP-index) was obtained by subtracting SNP-index of green bulk from SNP-index of virescent bulk. An average of SNP-index of SNPs located in a given genomic interval was calculated using a sliding window analysis with 1 Mb window size and 10 kb increment. The SNP-index graphs for virescent bulk and green bulk, as well as corresponding Δ (SNP-index) graph were plotted. Statistical confidence intervals of Δ (SNP-index) was calculated under the null hypothesis of no QTLs, and plotted them along with Δ (SNP-index) (Takagi et al., 2013).

### Fine Mapping Strategy

For confirming the result of BSA-seq analysis, linkage analysis was performed in the 185 F_2_-A plants which was the same segregating population for BSA-seq analysis. The markers on chromosome 3 were selected from the SSR library of ‘9930’ (Ren et al., 2009) and ‘Gy14’ (Cavagnaro et al., 2010). Then the polymorphic markers were screened between two parents and their F_1_ plant. The initial mapping of *v-2* was conducted in the 185 F_2_-A population by combining the genotypic with phenotypic data. Based on the result of initial mapping of *v-2*, 249 virescent plants in the F_2_-B population and 108 virescent plants in the BC_1_-A population were used to narrow down the genomic region of *v-2*. Furthermore, a larger F_2_-C population (n = 686) were applied to fine-map the *v-2* locus. During the fine mapping process, the InDels and SNPs markers were developed based on BSA-seq analysis described above. For SNP genotyping, the SNPs were directly conducted with Sanger sequencing in the recombinants.

### Comparative Transcriptome Analysis Between 104Y and EC1

The first true leaf at the top of 104Y and EC1 plant were harvested respectively. The first true leaf obtained from one plant was used as one biological replicate. Three 104Y plants and three EC1 plants were prepared for samples collection as three biological replicates each one. Then, the total six samples were immediately frozen in liquid nitrogen and stored at -80°C. Total RNA was extracted with TRIzol Reagent (Invitrogen, Carlsbad, CA, USA) according to the manufacturer’s instructions, and RNase-free DNase I was used to clean out DNA. Separate RNAs of six samples were constructed for RNA-seq. Six cDNA sequencing libraries were generated using a NEBNext^®^UltraTM RNA Library Prep Kit for Illumina^®^ (NEB, USA) following manufacturer’s protocol. The clustering of the index-coded samples was performed on a cBot Cluster Generation System using a TruSeq PE Cluster Kit v4-cBot-HS (Illumina) according to the manufacturer’s instructions. After cluster generation, the library preparations were sequenced on an Illumina Hiseq 2500 platform and paired-end reads were generated.

Clean data with high quality were obtained by removing reads containing adapters, reads containing ploy-N and low quality reads from raw data. After quality control with FastQC (http://www.bioinformatics.babraham.ac.uk/projects/fastqc/), the remaining high-quality reads were submitting for mapping analysis against the reference cucumber genome ‘ChiniseLong_V2’ (Huang et al., 2009) using Tophat v2.0.12. Following alignments, the number of reads mapped to each cucumber gene model was derived, and then normalized to fragments per kilobase of exon per million fragments mapped (FPKM). Transcripts with a minimal 2-fold difference in expression (|log_2_ FC (fold change)| ≥ 1) and an adjust false discovery rate (FDR) ≤ 0.05 were considered as differentially expressed genes (DEGs)

### Gene Annotation and Candidate Gene Identification

Fine mapping delimited the *v-2* locus into a 73-kb genomic DNA region on the cucumber chromosome 3. The numbers and functions of candidate genes in this target region were predicted using the cucumber genome database (ChiniseLong_V2) in the Cucurbit Genomics Databse (http://cucurbitgenomics.org/). For the identification of the candidate gene associated with *v-2*, the DNA and cDNA sequences of candidate genes in 104Y and EC1 were cloned and sequenced. The sequences alignments were conducted with the software DNAMAN to detect the variations. The expression levels of candidate genes located in the 73-kb genomic region were detected in the first true leaves of EC1 and 104Y by quantitative real-time PCR analysis (qRT-PCR).

## Results

### Characterization of the Virescent Mutant 104Y

**Figure 1:**
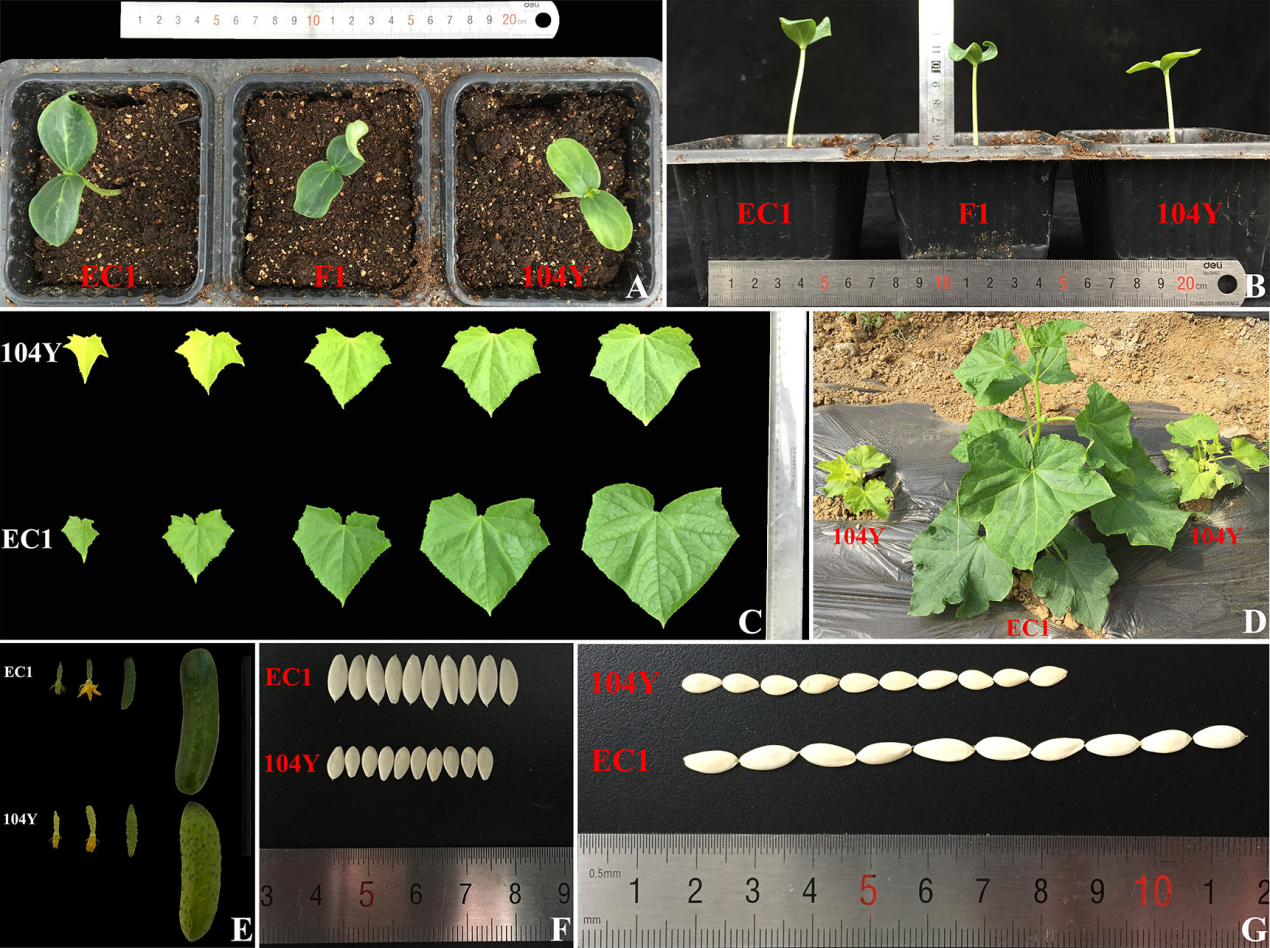
Phenotypic characterizations of virescent leaf mutant 104Y and normal green plant EC1. **(A)** The cotyledon color of 104Y and EC1 in the seedling stage. **(B)** The hypocotyl height of 104Y and EC1 at seedling stage. **(C)** The color change of true leaves from the first to the fifth true leaves in the 104Y and EC1. **(D)** The phenotypes of 104Y and EC1 plants at adult plant stage. **(E)** The color change of ovaries and fruits in 104Y and EC1. **(F)** The seeds width of 104Y and EC1. **(G)** The seeds length of 104Y and EC1.

**Figure 2:**
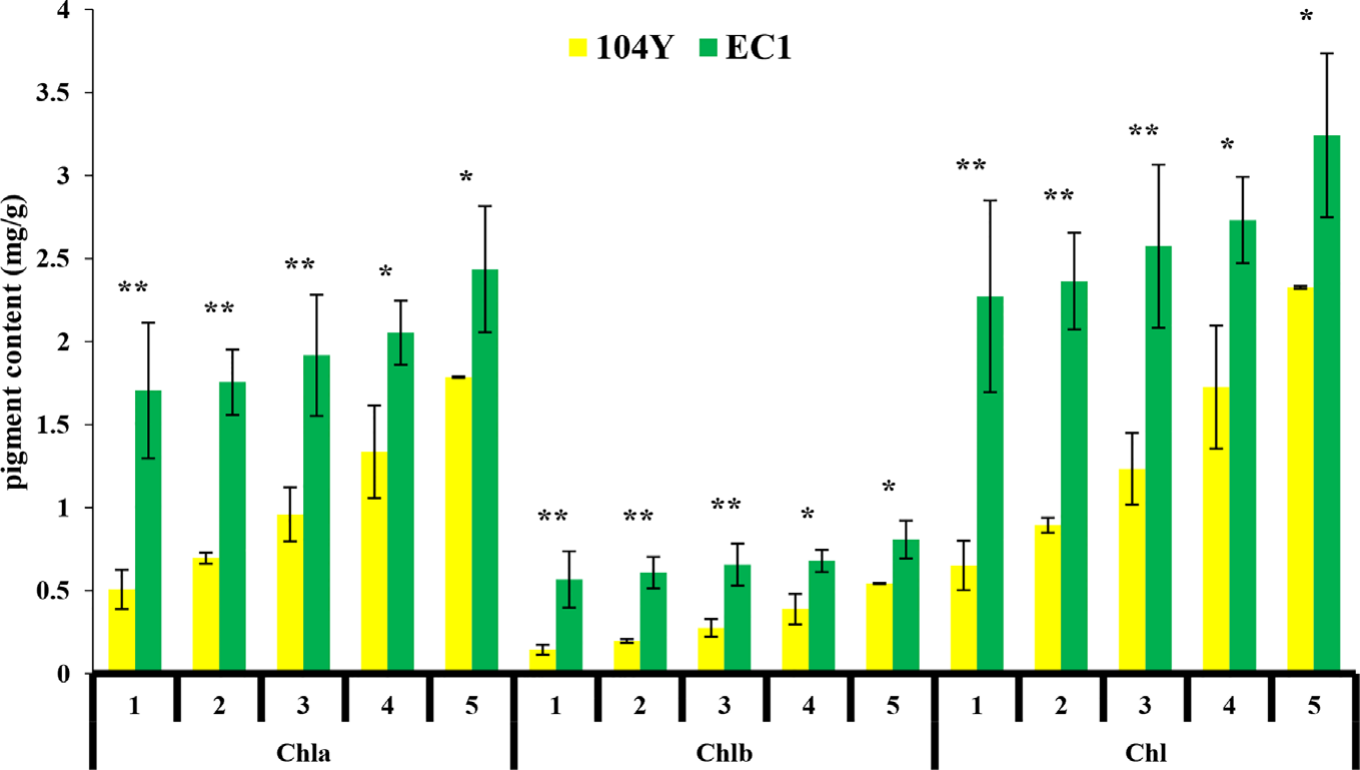
Chlorophyll a (Chla), Chlorophyll b (Chlb), and total chlorophyll (Chl) contents in milligrams per gram of fresh weight from the first true leaves to the fifth true leaves in 104Y and EC1. Data are mean ± SD (n = 3). Error bars represent SD of three independent replications. *, **: Significant at 5% and 1% level, respectively.

The virescent mutant 104Y was identified by natural mutation. The cotyledon of 104Y was yellow color when seedlings firstly emerged from the soil (**Figure 1A**) and gradually turned green following the true leaves grow up. The hypocotyl of the 104Y mutant was shorter than that of normal green plant (**Figure 1B**). The new young leaf of 104Y plant was yellow and gradually turned green as the plant growth up. The upper five true leaves of 104Y exhibited yellow-green color phenotype and gradually turned green from the first leaf to the fifth leaf (**Figure 1C**). The sixth leaf with normal green color occurred there-after. As compared with normal green plant EC1, chlorophyll (Chla, Chlb and Chl) contents were reduced in the 104Y from the first leaf to the fifth leaf, but were gradually increased following the position of the true leaf from top to bottom (**Figure 2**). The adult plant of 104Y was dwarf but fertile (**Figure 1D**). The ovaries and fruits of 104Y were greenish-yellow color (**Figure 1E**). The seeds of 104Y were smaller than that of normal green cucumber plant EC1 (**Figures 1F, G**). The growth rate and flowering time of 104Y plant were slower than those of normal green plant.

**Figure 3:**
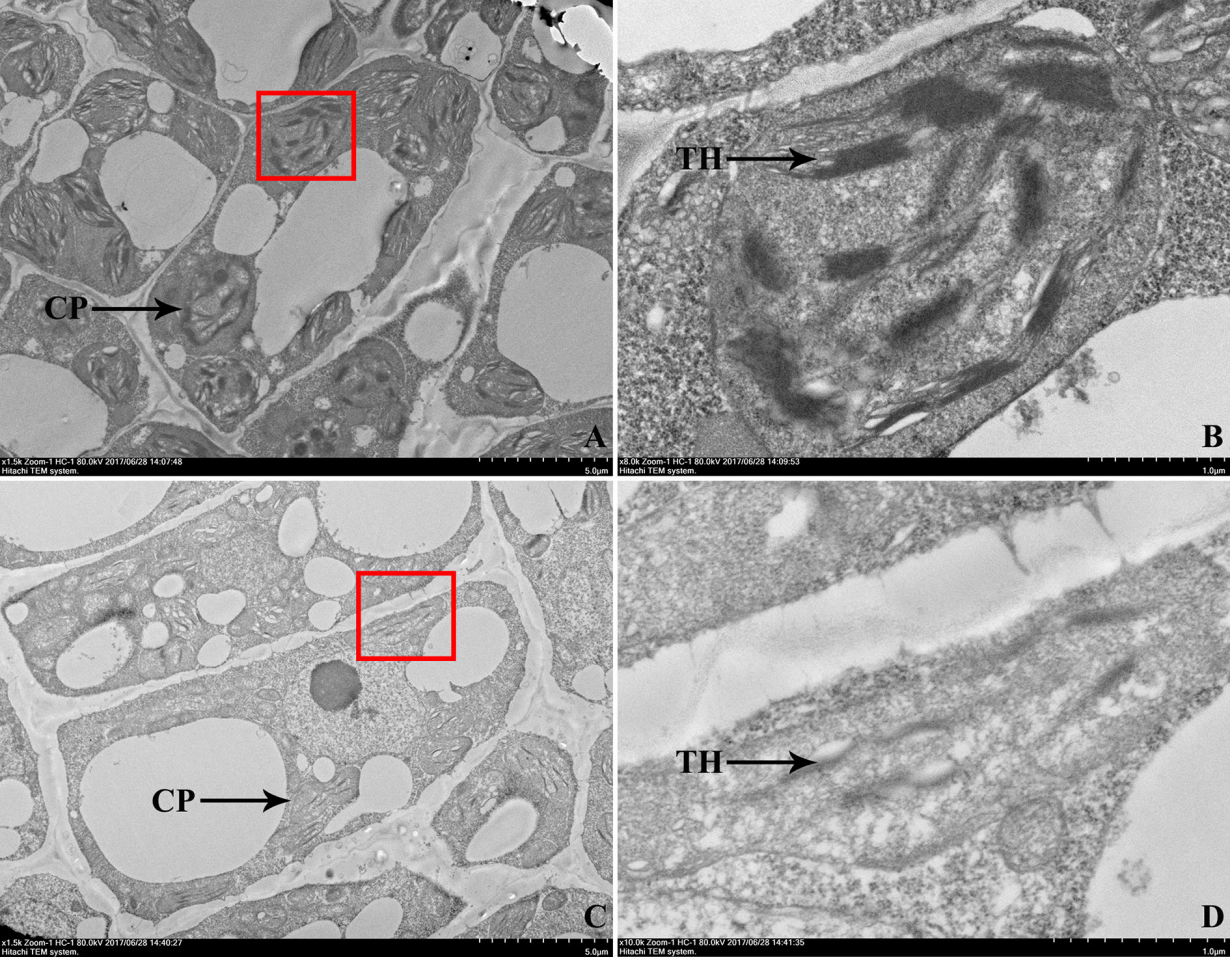
Transmission electron microscopic observation of the first true leaves in EC1 and 104Y. **(A, B)** Transmission electron microscopy of the chloroplast ultrastructure of EC1 leaf, **(B)** was a larger version of the red box in **(A)**. **(C, D)** Transmission electron microscopy of the chloroplast ultrastructure of 104Y leaf, **(D)** was a larger version of the red box in **(C)**. CP was the abbreviation of chloroplast. TH was the abbreviation of thylakoid.

To examine whether the leaf color deficiency was accompanied by the changes in chloroplasts, the ultrastructure of chloroplasts in the first leaves of 104Y mutant and normal green plant EC1 were compared by TEM observation, which were shown in **Figure 3**. As compared with EC1, 104Y exhibited fewer thylakoids per chloroplast. The results indicated that the chloroplast development in the virescent mutant 104Y was defective.

### Genetic Analysis of *v-2* Locus

To analyze the inheritance pattern of virescent leaf trait, the virescent mutant 104Y was crossed with green leaf plant EC1 to generate F_1_, F_2_ and BC_1_ populations. The F_1_ plant exhibited green leaf, similar to that of EC1. In the 185 F_2_-A population, there are 143 green leaf plants and 42 virescent leaf plants, fitting to 3:1 segregation ratio (*χ*^2^ = 0.521, *p* = 0.471). The phenotypic data on leaf color collected from the F_2_-B and F_2_-C populations were also accorded with the expected three green to one virescent segregating ratio in the *χ*^2^ test. For the 226 BC_1_-A plants derived from the cross between F_1_ plant and 104Y, 117 plants had green leaf and 108 plants had virescent leaf, corresponding to the 1:1 segregation ratio (*χ*^2^ = 0.36, *p* = 0.549). For the 87 BC_1_-B plants derived from the cross between EC1 and F_1_, they were all the green leaf color (**Table 1**). These results showed that the virescent leaf phenotype of 104Y mutant was controlled by one single recessive nuclear gene. According to the international rules for the naming of cucumber genes, and the *virescent* (*v*) and *virescent-1* (*v-1*) have been named (Miao et al., 2016; Weng and Wehner, 2017), thus, the virescent leaf mutant gene in this study was named as *virescent-2* (*v-2*).

**Table 1:** Segregation of plant leaf color in F_2_ and BC_1_ populations.

Populations	Pedigree	Segregation			Expected ratio (G:V)	*χ2*	*P* value in *χ2* test
		# Plants tested	Green plants	Virescent plants			
F_2_-A	F_1_(EC1×104Y)⊗	185	143	42	3:1	0.521	0.471
F_2_-B	F_1_(EC1×104Y)⊗	1092	843	249	3:1	2.813	0.093
F_2_-C	F_1_(EC1×104Y)⊗	686	514	172	3:1	0.002	0.965
BC_1_-A	F_1_(EC1×104Y)×104Y	226	117	108	1:1	0.360	0.549
BC_1_-B	EC1×F_1_(EC1×104Y)	87	87	0	–	–	–

### Initial Mapping of *v-2* Locus

**Figure 4:**
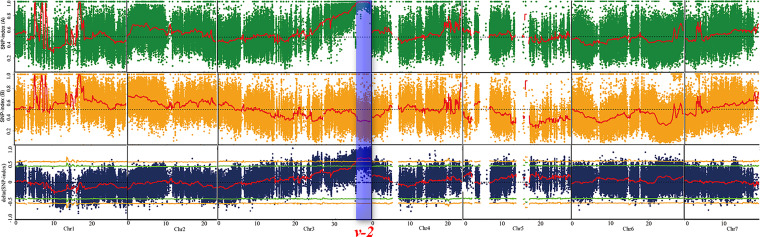
BSA-seq approach for mapping virescent leaf color locus. Red lines indicated the SNP-index graph, yellow lines indicated the probability values at 99% confidence (*P* < 0.01), green lines indicated probability values at 95% confidence (*P* < 0.05), blue box indicated the target genomic region controlling virescent leaf.

**Figure 5:**
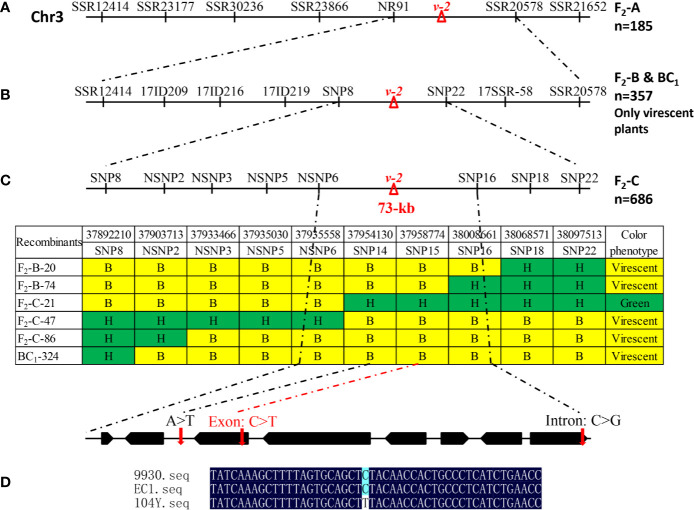
Fine mapping of virescent leaf color (*v-2*) locus. **(A)** Initial mapping with 185 F_2_-A plants placed *v-2* between NR91 and SSR0578. **(B)** Fine mapping with 249 virescent plants in the F_2_-B population and 108 virescent plants in the BC_1_ population narrowed down the *v-2* locus into 116 kb DNA region between markers SNP8 and SNP16. **(C)** Further fine mapping with 686 F_2_-C plants delimited *v-2* locus into 73 kb genomic region including eight genes. **(D)** As compared with EC1 and 9930, a single nucleotide substitution resulted in amino acid change in the gene *Csa3G890020* in the 104Y.

To identify the genomic region of virescent gene *v-2*, BSA-seq analysis was conducted on the 185 F_2_-A plants and identified only one significant locus (36.0-39.7 Mb) on chromosome 3 with a peak Δ (SNP-index) value of 0.8 (**Figure 4**). Based on the initial mapping result of BSA-seq, the linkage map of chromosome 3 was constructed with the 185 F_2_-A plants. Combining with the phenotypic data of 185 F_2_ individuals, linkage analysis detected that the *v-2* locus was located at 36.9-38.7 Mb region between markers NR91 and SSR20578 on the chromosome 3 (**Figure 5A**). These results of BSA-seq and linkage analysis validated with each other, and confirmed that the *v-2* locus was mapped to 36.9-38.7 Mb on chromosome 3.

### Fine Mapping and Candidate Gene Identification of *v-2* Locus

Initial mapping located the *v-2* locus between markers NR91 and SSR20578 on chromosome 3. To further narrow down the genomic region of *v-2*, 249 virescent plants in F_2_-B population and 108 virescent plants in BC_1_-A population were used. Based on the BSA-seq analysis, polymorphic markers including SSRs, InDels and SNPs in the 36.9-38.7 Mb of chromosome 3 were developed for fine mapping. Combining the genotypic data with the phenotypic data of the 357 virescent plants, the *v-2* locus was delimited into 205 kb DNA region between markers SNP8 and SNP22 (**Figure 5B**). Furthermore, another F_2_-C population with 686 individuals was used for fine mapping of *v-2*. Then, the *v-2* locus was further narrowed down into 73 kb DNA region with six recombinants (**Figure 5C**).

In the 73 kb genomic DNA region, eight genes were annotated using the ‘ChiniseLong_V2’ genome sequence data. The information and predicted functions of eight genes were presented in **Table 2**. Based on the BSA-seq analysis, five SNPs existed in the 73 kb genomic region (**Supplementary Table 1**). Among them, only one SNP (SNP15) existed in the exon resulting to nonsynonymous mutation of *Csa3G890020* gene (the 1895th base in the exon: C to T, the 632th amino acid in the protein: arginine to lysine) was detected in the virescent mutant 104Y (**Figure 5D** and **Supplementary Figure 1**). The other SNPs were detected in the intergenic, downstream or intronic regions. The SNP15 was also detected in the RNA-seq analysis between 104Y and EC1. Then, all the eight genes in the EC1 and 104Y were cloned and sequenced respectively. The results showed that the SNP15 was indeed detected in the *Csa3G890020* gene in the 104Y but not EC1. There were no differences in the cDNA sequences of the other seven genes in the 104Y and EC1. The *Csa3G890020* gene encodes an auxin F-box protein, which participated in the pathway of auxin signal transduction. While the auxin is connected with the regulatory network of chlorophyll biosynthesis, which means that the *Csa3G890020* gene was the most likely candidate gene for virescent leaf.

**Table 2:** Predicted genes in the 73 kb region of cucumber chromosome 3 harboring virescent gene *v-2*.

Gene No.	Gene ID	Position	Annotation
1	*Csa3G890000*	37940452-37940949	Unknown protein
2	*Csa3G890010*	37944417-37947213	Auxin F-box protein 5
3	*Csa3G890020*	37957798-37961366	Auxin F-box protein 5
4	*Csa3G890030*	37964780-37978058	WD and tetratricopeptide repeat protein
5	*Csa3G890040*	37982442-37986441	GF14 protein
6	*Csa3G890050*	37988001-37989974	Pentatricopeptide repeat-containing protein
7	*Csa3G890060*	37991530-37994560	Chaperone protein dnaJ
8	*Csa3G890070*	38000319-38009725	Unknown protein

**Figure 6:**
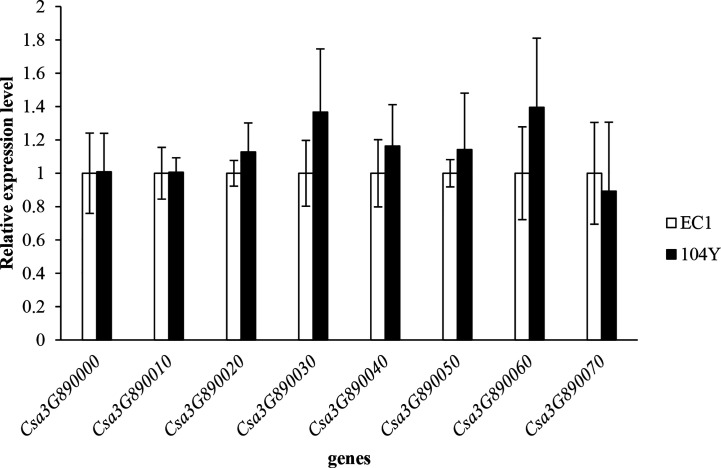
Relative expression levels of eight candidate genes in the first true leaf of EC1 and 104Y by qRT-PCR analysis. Data were displayed using the *CsActin* gene as an internal control with three biological and three technical replicates. Values are the mean ± SD.

Alignment of 14 homologous protein sequences from other plant species with *Csa3G890020* protein in cucumber revealed a high degree of sequence homology, varying from 66.83% (*Arabidopsis* homolog) to 92.12% (melon homolog). A phylogenetic tree was subsequently constructed using the neighbor-joining (NJ) method (**Supplementary Figure 2**). The *Csa3G890020* protein and melon transport inhibitor response 1-like protein, which showed the highest percentage of identity in the alignment, clustered together. Through qRT-PCR analysis, the expression levels of all the eight genes (the qRT-PCR primers of eight genes were shown in **Supplementary Table 2**) were not changed between 104Y and EC1 (**Figure 6**). The expression levels of *Csa3G890020* gene in the first, third, fifth and seventh true leaves of 104Y and EC1 were also not changed. Thus, we speculated the *Csa3G890020* gene performed the regulatory functions at post-transcriptional level rather than transcriptional level.

### Comparative Transcriptome Analysis Between 104Y and EC1

To reveal the possible molecular mechanisms of the formation of virescent leaf, RNA-seq analysis was performed with the first yellow true leaf of 104Y plant and the first green true leaf of EC1 plant. After RNA-seq analysis, the expression profiles of 15 cucumber genes (**Supplementary Table 3**) were performed by qRT-PCR to verify the accuracy of RNA-seq analysis. The results showed that the same expression patterns and the excellent Pearson’s correlations between qRT-PCR and RNA-seq data, which showed the high reliability of the RNA-seq results (**Supplementary Figure 3**).

**Figure 7:**
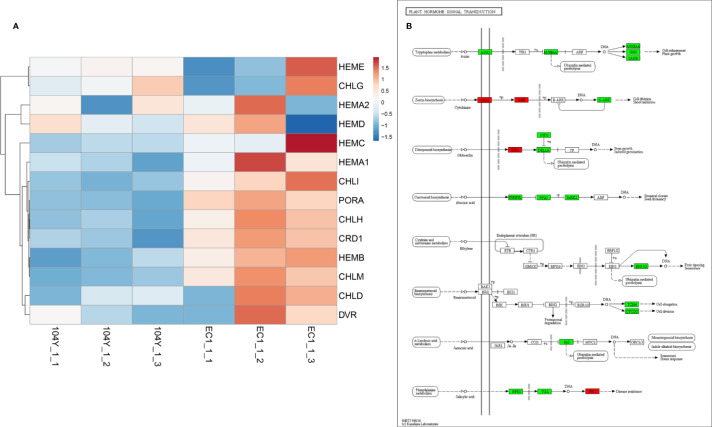
The expression levels of the genes involved in chlorophyll biosynthesis **(A)** and plant hormone signal transduction **(B)**.

In total, there were 2521 differentially expressed genes (DEGs) between the 104Y and EC1, among which 1235 and 1270 of the DEGs were up-regulated and down-regulated, respectively (**Supplementary Figure 4**). The GO annotations and functional classifications indicated that these DEGs were mainly enriched in iron ion binding in the molecular function category, photosystem I in the cellular component category and photosynthesis (movement of cell or subcellular component and microtubule-based movement were also enriched) in the biological process category (**Supplementary Figure 5**). The KEGG pathway enrichment analysis showed that these DEGs were mainly enriched in photosynthesis-antenna proteins (the highest rich value) and plant hormone signal transduction (the highest gene number) (**Supplementary Figure 6**). Compared with EC1-1, 10 DEGs in 104Y-1 were all down-regulated in the pathways of photosynthesis-antenna proteins. By processing RNA-seq data, the expression patterns of several key genes involved in chlorophyll synthesis were further analyzed. As compared with EC1, HEMC, HEMA1, CHLI, PORA, CHLH, CRD1, HEMB, CHLM and several key synthesis genes in the chlorophyll biosynthesis pathway were significantly down-regulated in the 104Y-1 (**Figure 7A**). As compared with EC1-1, 16 DEGs and 4 DEGs in 104Y-1 were down-regulated and up-regulated in the pathway of plant hormone signal transduction, respectively. Among them, four genes in the auxin signal transduction pathway were all down-regulated (**Figure 7B**). However, RNA-seq also detected no significant differences in the expression levels of the eight genes located in the *v-2* locus between 104Y and EC1 (**Supplementary Table 4**), which was same with the result of qRT-PCR analysis. The expression levels of Csa3G890020 were not changed from 1st to 7th true leaves between 104Y and EC1 (**Supplementary Figure 7**). Thus, we speculated that the *Csa3G890020* gene may affect the expression levels of other genes involved in the chlorophyll biosynthesis pathway or perform function in the protein level.

## Discussion

In cucumber, 17 leaf color mutants have been reported. In these leaf color mutants, only four virescent leaf mutants have been well characterized. The 9110Gt showed the yellow leaf color from the seedlings to the fourth true leaf stage (Miao et al., 2016). The adult plant of 9110Gt was all green leaves, which were similar with those of normal green cucumber plants. The *vyl* mutant exhibited virescent yellow leaf only in true leaves whereas the leaf vein remained green (Song et al., 2018). As compared with the normal green plants, 9110Gt and *vyl* mutant could grow up to the same or slightly smaller plant height. The *ygl1* mutant was a spontaneous mutant isolated from inbred cucumber 1402, which was slightly smaller than the wild type throughout the developmental stage (Ding et al., 2019). The C777 mutant displayed yellow color in cotyledons and true leaves with stay-green dots at emergence. The virescent leaf vein in C777 remained green color (Hu et al., 2020). In this study, the cotyledons and first to fifth true leaves including the leaf vein of 104Y were yellow in color. As the 104Y plant grow up, the yellow leaf gradually turn green, and the whole plant shows a gradual transition from yellow to green from top to bottom. At the adult plant stage, the plant height of 104Y mutant was significantly smaller than that of the normal green plants. From the phenotypic characterizations, the virescent leaf mutant 104Y in this study was different with the above four virescent leaf mutants. Thus, the 104Y mutant was believed to be a new virescent leaf mutant in cucumber.

In this study, the virescent leaf mutant 104Y was crossed with normal green cucumber plant EC1 to generate the F_1_, BC_1_ and F_2_ populations. The leaves of F_1_ plant were green color. The green and yellow color leaves in the BC_1_ and F_2_ populations were in accordance with the 1:1 and 3:1 Mendelian inheritance laws, respectively. The results indicated that the virescent leaf trait in 104Y was controlled by one single recessive nuclear gene. The virescent leaf trait had also been reported to be controlled by one single recessive nuclear gene in some other plants, such as *Arabidopsis thaliana* (Ma et al., 2017; Huang et al., 2018), rice (Wang et al., 2016; Zhu et al., 2016), cotton (Zhu et al., 2017; Mao et al., 2019), maize (Xing et al., 2014), cabbage (Liu et al., 2016), apple (Fernández-Fernández et al., 2014), watermelon (Zhang et al., 1996) and so on. In cucumber, the virescent traits in 9110Gt (Miao et al., 2016), *vyl* mutant (Song et al., 2018), *ygl1* (Ding et al., 2019) and C777 (Hu et al., 2020) were also controlled by one single recessive nuclear gene. It indicated that the virescent mutants were easy to form because the mutation of anyone gene involving in the pathways of chloroplast development or chlorophyll biosynthesis could generate the virescent leaf appearance (Beale, 2005).

BSA-seq method can be applied to rapidly identify specific genomic regions and candidate genes for a given trait (qualitative or quantitative) from crops with assembled genomes, such as rice (Takagi et al., 2013), cotton (Zhu et al., 2017), chickpea (Singh et al., 2016a), pigeonpea (Singh et al., 2016b), groundnut (Pandey et al., 2017), pea (Xue et al., 2017), tomato (Illa-Berenguer et al., 2015), pakchoi (Wang et al., 2018), cucumber (Zhang et al., 2018) and so on. BSA-seq can also be used to develop the different kinds of markers (SSR, InDel and SNP) with high polymorphic rate for fine mapping of the target gene. In this study, the virescent gene *v-2* was quickly mapped into 36.0-39.7 Mb on chromosome 3 by using BSA-seq analysis. The accuracy of BSA-seq analysis was then confirmed with linkage analysis. With BC1, another F_2_ populations and polymorphic markers (SSR, InDel, SNP) developed by BSA-seq analysis, the *v-2* gene was further narrowed into a ~73 kb region which contained eight genes. BSA-seq analysis identified three SNPs in the 73 kb genomic region. Among the three SNPs, one SNP (SNP14) occurred in the intergenic region between *Csa3G890010* and *Csa3G890020*, one nonsynonymous SNP (SNP15) occurred in the exon of *Csa3G890020*, one SNP (SNP16) occurred in the intron of *Csa3G890070*. The three SNP mutations in 104Y were also detected by comparative transcriptome analysis between 104Y and EC1. With DNA and cDNA sequencing of the eight genes in the 73 kb genomic region, SNP15 and SNP16 existed in the exon of *Csa3G890020* and intron of *Csa3G890070*, respectively. Since the nonsynonymous mutation occurring in the exon of gene would change the function. Thus, the *Csa3G890020* gene was predicted as the most likely candidate gene for virescent leaf.

In normal plant, auxin regulates transcription by stimulating the degradation a family of transcriptional repressor called Aux/IAA proteins. The Aux/IAA proteins exert their effects by binding to the ARF (auxin response factor) proteins through a conserved dimerization domain. In the presence of auxin, the auxin F-box protein TIR1/AFBs binds to the Aux/IAA proteins, resulting in their ubiquitination and degradation. Thus the degradation of Aux/IAAs leads to the derepression of ARF-mediated transcription. AFB genes have been suggested to play a key role in regulating the expression of auxin response genes (Liscum and Reed, 2002). In this study, the *Csa3G890020* gene encoding an auxin F-box protein was identified as a candidate gene for virescent leaf. Mutation in the *Csa3G890020* gene would leads to the unable interaction with Aux/IAA proteins. Thus, the Aux/IAA protein could continue to bind the ARF proteins, resulting in the breakdown of the auxin-dependent transcriptional regulation in plants. Previous studies have reported that auxin signaling was involved in the regulation of chlorophyll biosynthesis (Liu et al., 2017). Yuan et al. (2018) reported that *SlARF10* was involved in chlorophyll accumulation during tomato fruit development. Shen et al. (2015) reported that *OsARF16* had a role in regulating auxin transport and Fe homeostasis. The Fe homeostasis is essential for the chlorophyll biosynthesis (Jeong and Guerinot, 2009). In this study, the iron ion binding in the molecular function category was the most enrich in the GO enrichment analysis (**Supplementary Figure 5**). The expression levels of the genes involved in auxin signaling transduction network were all down-regulated. Thus, the lower expression levels of the genes involved in chlorophyll biosynthesis and auxin signaling transduction network were resulted in the formation of virescent leaf phenotype, slow growth, delayed flowering, dwarf in 104Y. The regulation of TIR1/AFB-Aux/IAA-ARF complex interacted with each other, no matter which one was mutated, the other two were affected in the transcription levels. From RNA-seq data, the mutation in the *Csa3G890020* gene did not change its expression level, but the expression level of Aux/IAA was down-regulated. The expression level of ARF gene was not changed, but its downstream regulatory genes were all down-regulated (**Figure 7B**). It indicated that the regulatory functions of AFB and ARF genes were not performed at transcriptional level but post-transcriptional level. Thus, we speculated that the auxin signaling transduction network was changed by the mutation in AFB gene, which regulated the chlorophyll biosynthesis, resulting in the formation of the virescent leaf. This may be a new regulatory mechanism in leaf color variation. However, whether the mutation in the *Csa3G890020* protein will affect the formation of TIR1/AFB-Aux/IAA-ARF complex. This query will be further validated with the western blotting analysis of *Csa3G890020* protein between virescent mutant 104Y and normal green plant EC1. We will also identify the interaction protein of *Csa3G890020* protein by the verification of yeast two-hybrid assays and bimolecular fluorescence complementation.

## Data Availability Statement

The raw data from BSA-seq analysis have been deposited into the Sequence Read Archive (https://www.ncbi.nlm.nih.gov/sra/) with accession number SRP252648. The clean data from comparative transcriptome analysis were deposited in the Sequence Read Archive under accession number SRP252797.

## Author Contributions

JC and JL designed and supervised the experiments. KZ, YW, and MN collected phenotypic data for virescent leaf. KZ, YL, and WZ performed the linkage analysis of virescent leaf gene. KZ, QL, JL, and JC contributed to revising the manuscript. All authors contributed to the article and approved the submitted version.

## Funding

This research was supported by National Key Research and Development Program of China (2016YFD0101705-5, 2016YFD0100204-25, 2018YFD1000804), National Natural Science Foundation of China (No. 31672168) and Jiangsu Agricultural Innovation of New Cultivars (No. PZCZ201719).

## Conflict of Interest

The authors declare that the research was conducted in the absence of any commercial or financial relationships that could be construed as a potential conflict of interest.
