# Supplementary material for: Fine Mapping and Transcriptome Analysis of Virescent Leaf Gene v-2 in Cucumber (Cucumis sativus L.)
Source: Front Plant Sci. 2020 Sep 25;11:570817. doi: 10.3389/fpls.2020.570817 (PMC7545910; doi:10.3389/fpls.2020.570817)
Supplement: Supplementary file 1 [file DataSheet_1.pdf]

Supplementary Material

1 Supplementary Figures

MRVDKAEMSEDEDRAQSLDLGIEAAAESANKTRNCSGGDGEEVGGSGSVENILHNVLEN
VLHFLTSRRDRNAASLVCKSWYRVEALTRSELFIGNCYAVSPRRVTSRFSRVRSVSIKGKPR
FADFNLMPHNWGAHFTPWVAAMAKSYPWLERVYLKRMSVTDDDLALLADSFPGFKELV
LFCCEGFGTSGIAVVAARCRHLRVLDLIASDVGDDEVDWISCFPEKETCLESLIFECVEWPI
NFEALERLVSRSPSLKKLGVNRHVSIAQLYQLMIRAPRLTHLGTGSFNTLEAVIHGESEPDY
ASVFAACNSLVCLSGFKDVLPDYLPCIYPVCANLTTLNLSYANITPEQLKPAIRHCHKLQTF
WALDSICDEGLQAVASTCKELRELRVFPFDAREDVEGPISEVGFQAISEGCRKLQYILYFCQ
RMTNAAVVAMSQNCQDLVVFRLCIMGRHQPDHKTGDPMDEGFGAIVINCKKLTRLAISG
LLTDRAFSYIGKYGKLVRTLSVAFAGNSDLGLKYVLEGCHRLQKLEIRDSPFGDIALHSGL
HHYYNMRFLWMSDCKLSRGGCQEVAKAMPHLVVEAMRNEIEEVDYLPQVEDLDNHVR
LLYMYRSLEGPRDDAPEFRGSDEGSGC(R/K)AALKALITSESLVIHRIKIELENVGDLINYK
RSKNIVLSLSSSKVREWLQGSLQASISGLYFSLLREKQALLASKEGGKSNGIIKVGSDCASV
ALNEKLRLCFLV*

Supplementary Figure 1. The 632th amino acid substitution in the *Csa3G890020* protein. R is the arginine (the normal amino acid), K is lysine (the mutant amino acid).

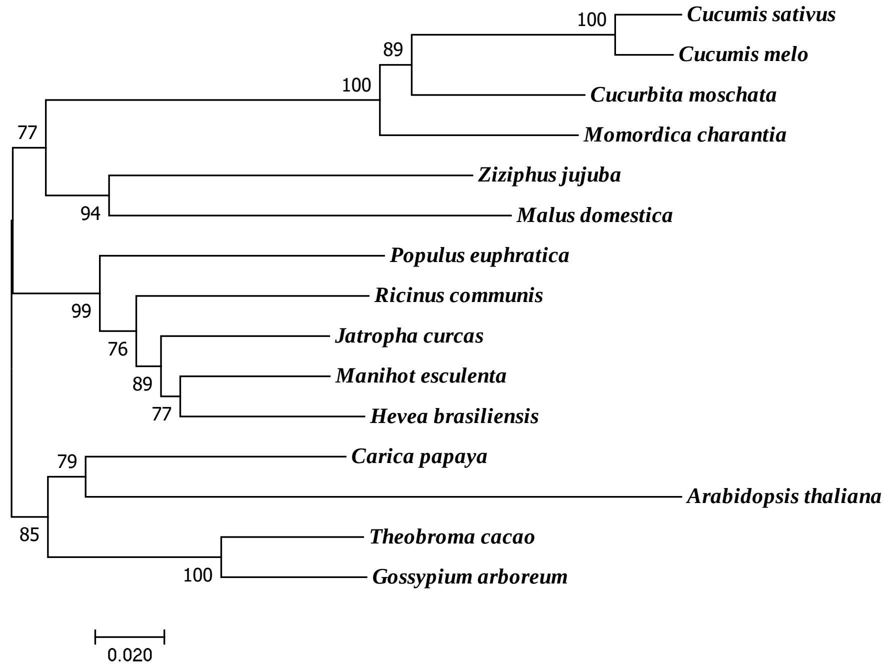

Supplementary Figure 2. Phylogenetic tree of closely related homologs of Auxin F-box proteins in cucumber and other species. The phylogenetic tree was constructed using the neighbor-joining method built in MEGA 7.0. The numbers indicate the percentage of replicate trees in which the associated taxa clustered together according to the bootstrap test (1000 replicates).

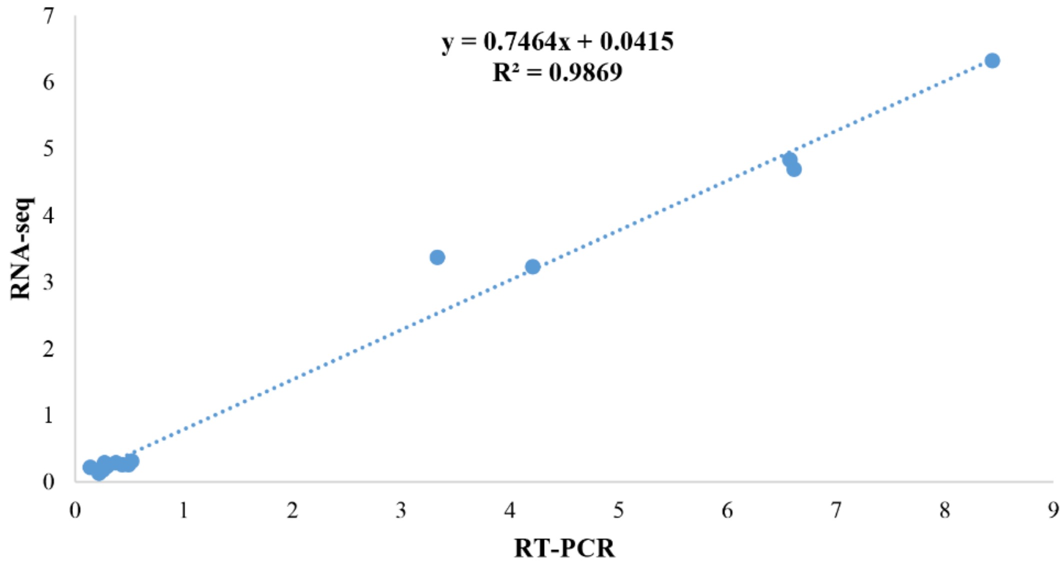

Supplementary Figure 3. Verification of the expression profiles of 15 genes obtained in RNA-seq analysis by qPCR. The RNA-seq Fold-change value of 104Y/EC1 (y-axis) was plotted against the value from the RT-PCR (x-axis).

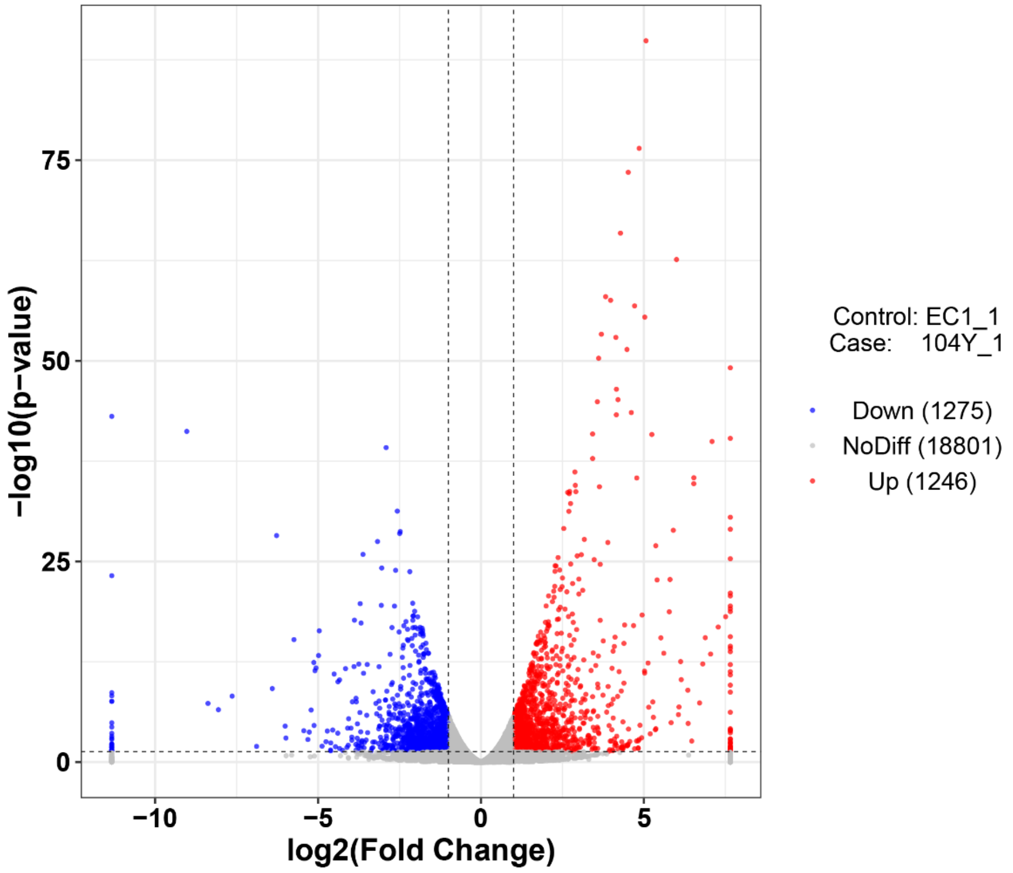

Supplementary Figure 4. The differentially expressed genes (DEGs) between the first true leaves of 104Y and EC1.

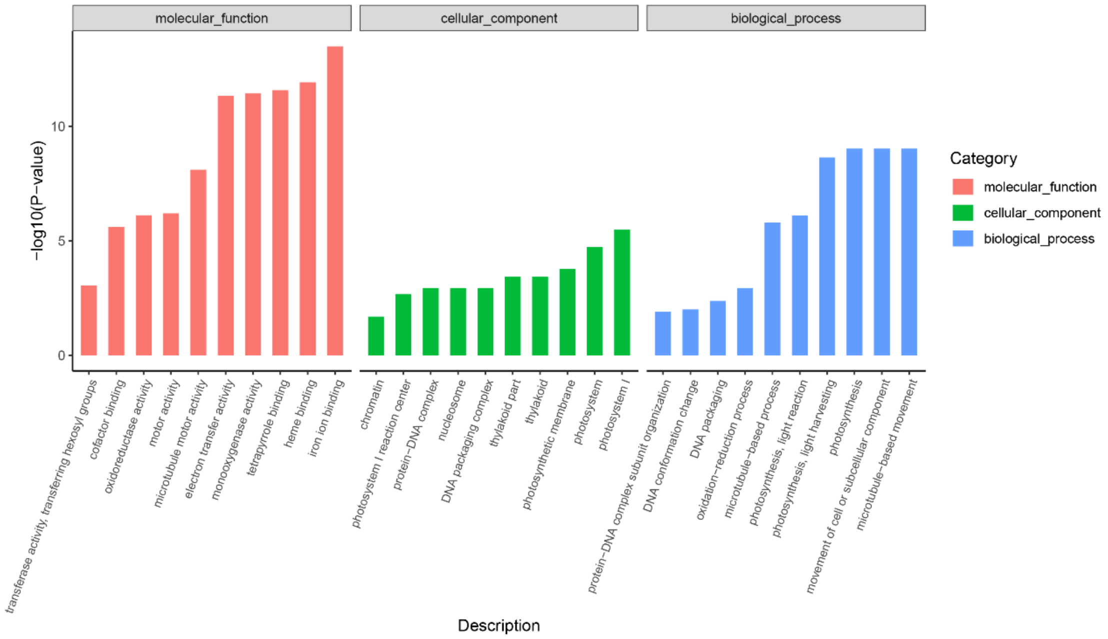

Supplementary Figure 5. GO annotations and functional classifications of the DEGs between 104Y and EC1.

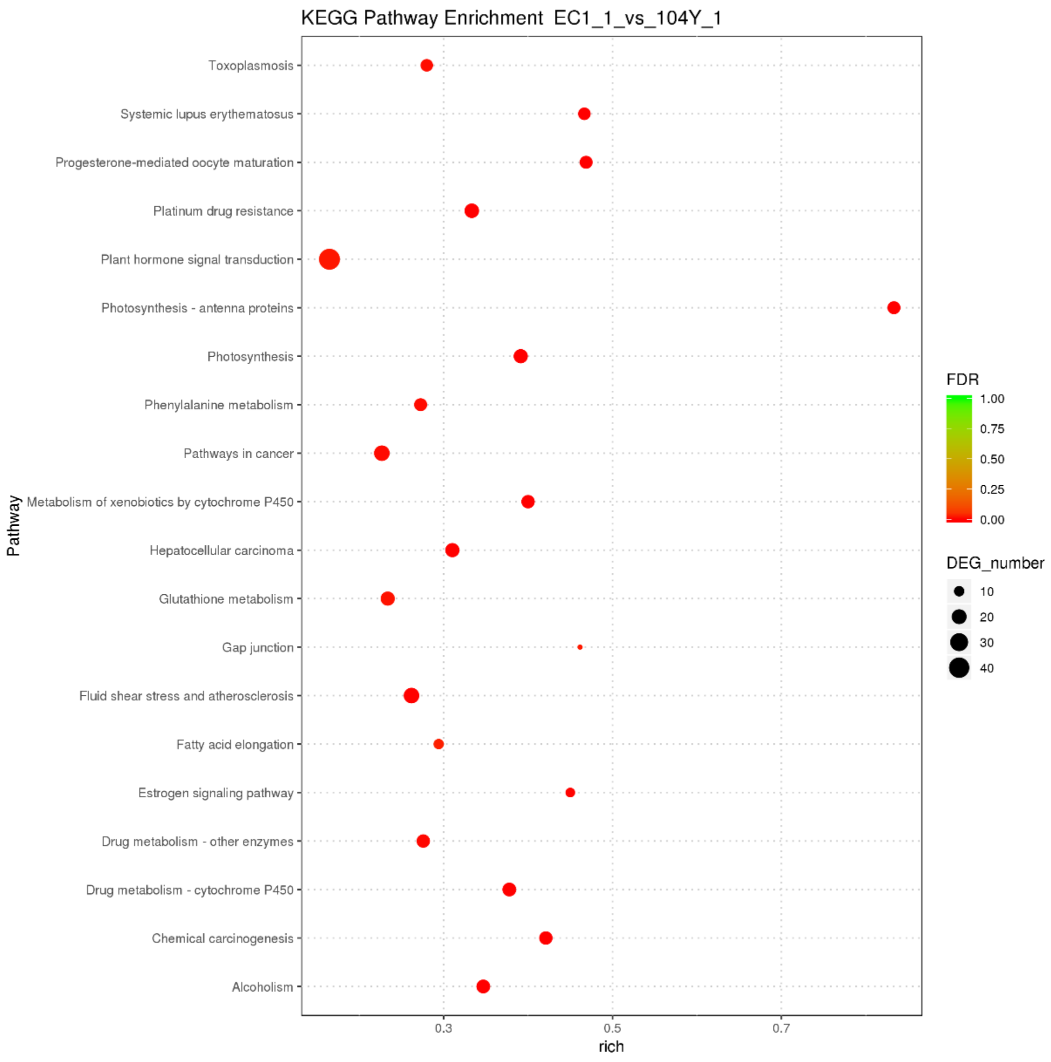

Supplementary Figure 6. KEGG pathway enrichment analysis of the DEGs between 104Y and EC1.

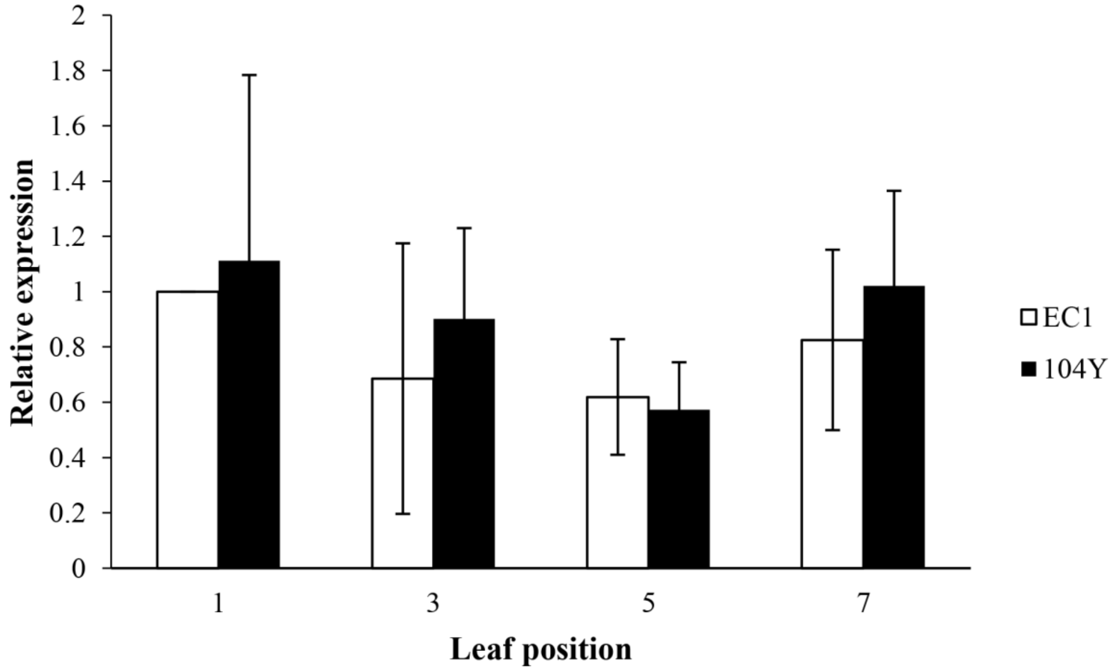

Supplementary Figure 7. Relative expression levels of Csa3G890020 gene in the first, third, fifth and seventh true leaves of EC1 and 104Y by qRT-PCR analysis. Data were displayed using the *CsActin* gene as an internal control with three biological and three technical replicates. Values are the mean ± SD.

2 Supplementary Tables

Supplementary Table 1. The information of SNP mutation in the *v-2* locus based on BSA-seq analysis.

<table>
<tr><th>Chr</th><th>Pos</th><th>Ref</th><th>Alt</th><th>EC1_ genotype</th><th>EC1_ ref_alt_depth</th><th>104Y_ genotype</th><th>104Y_ ref_alt _depth</th><th>Green_ bulk_ genotype</th><th>Green _bulk_ ref_alt _depth</th><th>Virescent _bulk_ genotype</th><th>Virescent_ bulk_ref_ alt_depth</th><th>Region</th><th>Gene</th><th>Feature</th></tr>
<tr><td>Chr3</td><td>37935558</td><td>T</td><td>C</td><td>0@0</td><td>26|0</td><td>1@1</td><td>0|32</td><td>0@1</td><td>17|5</td><td>1@1</td><td>0|30</td><td>intergenic</td><td>Csa3G889990 (dist=2167), Csa3G890000 (dist=4894)</td><td>-</td></tr>
<tr><td>Chr3</td><td>37941105</td><td>A</td><td>G</td><td>0@0</td><td>18|0</td><td>1@1</td><td>0|40</td><td>0@1</td><td>22|11</td><td>1@1</td><td>0|22</td><td>downstream</td><td>Csa3G890000</td><td>-</td></tr>
<tr><td>Chr3</td><td>37954130</td><td>A</td><td>T</td><td>0@0</td><td>24|0</td><td>1@1</td><td>0|40</td><td>0@1</td><td>18|4</td><td>1@1</td><td>0|19</td><td>intergenic</td><td>Csa3G890010 (dist=6917), Csa3G890020 (dist=3668)</td><td>-</td></tr>
<tr><td>Chr3</td><td>37958774</td><td>C</td><td>T</td><td>0@0</td><td>18|0</td><td>1@1</td><td>0|37</td><td>0@1</td><td>20|9</td><td>1@1</td><td>0|24</td><td>exonic</td><td>Csa3G890020</td><td>nonsynonymous SNV</td></tr>
<tr><td>Chr3</td><td>38008661</td><td>C</td><td>G</td><td>0@0</td><td>20|0</td><td>1@1</td><td>0|25</td><td>0@1</td><td>28|11</td><td>1@1</td><td>0|34</td><td>intronic</td><td>Csa3G890070</td><td>-</td></tr>
</table>

Supplementary Table 2. List of primers used in quantitative RT-PCR for the 8 genes located in *v-2* locus.

**Gene ID**	**Forward primer 5' to 3'**	**Reverse primer 5' to 3'**
Csa3G890000	GTTGTGCGAGAGTGGAGGTTA	GCGACTTGGGTGATGAGCAA
Csa3G890010	GAACGCGGCTTCACTTGTTT	GCGACGTAACTCTACGAGGG
Csa3G890020	TCGTGGTATCGTGTTGAGGC	CTCGACTAAACCGCGACGTA
Csa3G890030	TGGAGGACTTCGCCTTTCAC	GCATCTGCAAACCATAGGCG
Csa3G890040	GGCGCATCGTATCCTCCATT	AGACCTCGGTGAGTTCGGAT
Csa3G890050	CTTGGGATGAAGCCCGATACT	CGGCTCTGCAATCTTTGTCC
Csa3G890060	TCGACAACCCGACTCCGATA	TGTCATCGTCGGTAGCATCG
Csa3G890070	CATGGCATATGTGCACGCAA	ACGGGTACGTTTCTTGGACC

Supplementary Table 3. List of primers used in quantitative RT-PCR for validation of RNA-seq analysis.

Gene ID	Forward primer 5' to 3'	Reverse primer 5' to 3'
Csa1G126040	ATATGAAGCGTCAAACCGCA	TAGCGTTCGTTAAGCAGACC
Csa1G153560	TCGGGATATTAGGGACTGGAA	TACTATCCCGCCGATCGTT
Csa3G099680	GAGTTAAGTACTTGGGGCCG	ACCTTCCGTGGATTACCTCA
Csa3G145780	CATTCTGTCAACCACCGCTA	TGATGGAATCGCTTGCTCTC
Csa3G826660	CACAATACCACAAGCCATGC	GACAAACGGCTTTGTTTCCA
Csa4G152270	GAAGTGTTAGAAAGGCTGCG	CGGTTGTTGTGAGGAGGATTA
Csa5G153020	ATGATCTTCGCCCTCGTCTA	TACTGCTCTTGTGAGGGACA
Csa5G623470	CCAACCATTTTCAACGACCG	CTGCCATGCTGATGTTTGTG
Csa6G076800	AATGGCGCTCTCTCTCCTAT	CCAAGACGATGTGGAGACTG
Csa6G139750	ACCCCCATCCTATCTTACCG	GCCCATCTCGAGTGGATTAC
Csa6G157120	GAACCGGTTGTCATCAGGAA	AGATTTGTTCGACCCAGCAG
Csa6G423360	ATTCTATGGGACCCCACCAA	TACCAAAAGCACTGCCCATT
Csa6G505950	GACTCATCTGCCACCGATTT	AGCTTCCGATCGTAATCAGC
Csa6G522690	CCAAGAACCGTGAGTTGGAA	TCACACCATTGCGAGACAAA
Csa7G351890	TTTAATGGAGGTGGGTTGGC	CAGAATGTTTGGTTGTCGGC

Supplementary Table 4. The FPKM values of the 8 genes located in *v-2* locus based on RNA-seq analysis.

Gene ID	104Y_1_1	104Y_1_2	104Y_1_3	EC1_1_1	EC1_1_2	EC1_1_3
Csa3G890000	7.6387987	6.6341431	8.2346802	6.3649471	7.1188034	5.1002666
Csa3G890010	13.524667	14.525304	15.730124	18.800003	19.978988	18.742411
Csa3G890020	9.4083245	8.5075366	9.644823	12.249818	11.159044	9.4676353
Csa3G890030	3.7960014	3.3385003	3.2211876	2.370509	2.4131446	2.1350157
Csa3G890040	37.337254	34.668195	36.612307	39.262073	36.772474	30.671381
Csa3G890050	5.2156447	4.0054429	4.674527	4.3022805	3.1570973	4.3262344
Csa3G890060	3.7424076	3.9554284	4.128211	5.6899056	4.2430888	4.4389873
Csa3G890070	6.79787	8.6410627	8.7266949	11.714972	7.7295704	8.068231
